# Antitussive, Antioxidant, and Anti-Inflammatory Effects of a Walnut (*Juglans regia* L.) Septum Extract Rich in Bioactive Compounds

**DOI:** 10.3390/antiox10010119

**Published:** 2021-01-15

**Authors:** Ionel Fizeșan, Marius Emil Rusu, Carmen Georgiu, Anca Pop, Maria-Georgia Ștefan, Dana-Maria Muntean, Simona Mirel, Oliviu Vostinaru, Béla Kiss, Daniela-Saveta Popa

**Affiliations:** 1 Department of Toxicology, Faculty of Pharmacy, Iuliu Hatieganu University of Medicine and Pharmacy, 8 Victor Babes, 400012 Cluj-Napoca, Romania; ionel.fizesan@umfcluj.ro (I.F.); anca.pop@umfcluj.ro (A.P.); stefan.georgia@umfcluj.ro (M.-G.Ș.); kbela@umfcluj.ro (B.K.); dpopa@umfcluj.ro (D.-S.P.); 2 Department of Pharmaceutical Technology and Biopharmaceutics, Faculty of Pharmacy, Iuliu Hatieganu University of Medicine and Pharmacy, 8 Victor Babes, 400012 Cluj-Napoca, Romania; rusu.marius@umfcluj.ro (M.E.R.); dana.muntean@umfcluj.ro (D.-M.M.); 3 Department of Pathological Anatomy, Faculty of Medicine, Iuliu Hatieganu University of Medicine and Pharmacy, 8 Victor Babes, 400012 Cluj-Napoca, Romania; 4 Department of Medical Devices, Faculty of Pharmacy, Iuliu Hatieganu University of Medicine and Pharmacy, 8 Victor Babes, 400012 Cluj-Napoca, Romania; smirel@umfcluj.ro; 5 Department of Pharmacology, Physiology and Physiopathology, Faculty of Pharmacy, Iuliu Hatieganu University of Medicine and Pharmacy, 8 Victor Babes, 400012 Cluj-Napoca, Romania

**Keywords:** walnut, by-products, antitussive, ROS, NOx, IL-6, CXC-R1, histopathological analysis

## Abstract

The antitussive, antioxidant, and anti-inflammatory effects of a walnut (*Juglans regia* L.) septum extract (WSE), rich in bioactive compounds were investigated using the citric acid aerosol-induced cough experimental model in rodents. Wistar male rats were treated orally for three days with distilled water (control), codeine (reference), and WSE in graded doses. On the third day, all rats were exposed to citric acid aerosols, the number of coughs being recorded. Each animal was sacrificed after exposure, and blood and lung tissue samples were collected for histopathological analysis and the assessment of oxidative stress and inflammatory biomarkers. The results of the experiment showed a significant antitussive effect of WSE, superior to codeine. This activity could be due to cellular protective effect and anti-inflammatory effect via the stimulation of the antioxidant enzyme system and the decrease of IL-6 and CXC-R1 concentration in the lung tissue of WSE-treated animals. The antioxidant and anti-inflammatory effects of WSE were confirmed by biochemical assays and histopathological analysis. This is the first scientific study reporting the antitussive effect of walnut septum, a new potential source of non-opioid antitussive drug candidates, and a valuable bioactive by-product that could be used in the treatment of respiratory diseases.

## 1. Introduction

Natural products such as endogenous or exogenous metabolites of animals and plants, played a significant role in the discovery of medicines as more than half of the medicines in use were developed from natural products [1]. Famous examples are aspirin, a product derived from salicylic acid, present in the bark of several species of genus *Salix* [2], codeine, a naturally occurring alkaloid found in plants of *Papaver* spp. [3], quinine, a component of the bark of trees in the genus *Cinchona* [4], or penicillin, the first antibiotic compound isolated from the fungus *Penicillium* [5]. The isolation and use of bioactive compounds from natural products can mitigate the high cost of chemical synthetic drugs in the development of new medications.

Cough is a defensive reflex often present in pulmonary diseases including asthma, chronic bronchitis, pulmonary neoplasm, and upper respiratory tract infections. Chronic cough is associated with significant morbidity such as exacerbation of asthmatic symptoms, breathlessness, rib fractures, pneumothorax, or syncope. The commonly used treatment can include administration of antitussive medication, codeine being one of the most prominent. Codeine has been prescribed as an analgesic or antitussive for many decades, but clinical evidence emphasized the potential danger of this compound. Codeine is metabolized into morphine in the liver, which is partially responsible for its analgesic and antitussive effects. However, the individual patient response to codeine differs greatly in children, with documented cases of unanticipated respiratory depression and death. Various regulatory bodies, including the US Food and Drug Administration and European Medicines Agency issued warnings regarding the occurrence of the negative effects of codeine in this age group. Alternative therapies are needed to prevent future opioid problems especially in pediatric patients [6].

It was already revealed that some macro‑ and micronutrients contained in tree nuts have favorable health benefits via different mechanisms of action [7,8]. Walnuts are considered nutraceuticals, as they contain proteins and essential unsaturated fatty acids, as well as tocopherols, sterols, and polyphenols with recognized antioxidant, anti-inflammatory, and antibacterial properties [9]. In addition to kernel, walnut by-products including leaves, green husk or septum represent important sources of nutraceutical compounds [10,11].

Walnut septum (WS), rich in polyphenolic compounds, demonstrated hypoglycemic effect, antioxidant and antimicrobial activity, hematopoiesis-stimulating potential, or anti-aging effects, both in vitro and in vivo [12,13]. A previous study on Wistar rats demonstrated that WS extract (WSE) had no subacute or acute toxic effects at doses of 1000 mg/kg body weight (b.w.) [14].

In folk medicine, WS is used along with hard walnut shell in decoctions to obtain an antitussive effect, but no scientific research supporting this claim has been published so far. Hence, in this study we planned to investigate the antitussive effects of a WSE obtained in optimum extraction conditions with rich polyphenolic composition [15] using a citric acid aerosol-induced cough rodent model [16,17]. This research could lead to the development of new non-opioid antitussive drug candidates of natural origin, potentially useful in a variety of respiratory diseases.

## 2. Materials and Methods

### 2.1. Reagents

All reagents and standards were of analytical grade. Acetone, Folin–Ciocâlteu (FC) reagent, hydrochloric acid, hematoxylin-eosin, paraffin were acquired from Merck (Darmstadt, Germany). Sodium carbonate, sodium chloride, sodium nitrite, acetic acid, disodium hydrogen phosphate, potassium dihydrogen phosphate, bovine serum albumin (BSA), Coomassie Brilliant Blue G (CBB), tris(hydroxymethyl)aminomethane (Tris), 2,2′-azinobis-(3-ethylbenzothiazoline-6-sulfonate) (ABTS), 6-hydroxy-2,5,7,8-tetramethyl chroman-2-carboxylic acid (Trolox), 2′,7′-dichloro-dihydro-fluorescein diacetate (DCFH-DA), phosphate-buffered saline (PBS), vanadium (III) chloride, sulphanilic acid, and alpha-naphthylamine were bought from Sigma-Aldrich (Schnelldorf, Germany). The normal saline solution (0.9% sodium chloride) was from B. Braun Melsungen AG (Melsungen, Germany). Neutral buffered formalin was obtained from Chempur (Lodz, Poland), codeine phosphate from Terapia S.A. (Cluj-Napoca, Romania). The water used in our study was ultrapure obtained from a Milli-Q ultrapure water system (Bucharest, Romania).

### 2.2. Preparation of the Walnut Septum Extract

Walnut septum was obtained from walnuts harvested in Maramureș County, Romania, in the fall of 2018. The previously identified optimal extraction conditions [15] were used to make the WSE with the highest total phenolic content (TPC). Briefly, WS was weighed (0.5 g) and mixed with 5 mL water: acetone (50:50, *v*/*v*) as extraction solvent. Turbo-extraction was applied as extraction method, first using an Ultra-Turrax homogenizer (T 18; IKA Labortechnik, Staufen, Germany) for 2 min (1 min at 9500 rpm and 1 min at 13,500 rpm), then a Vortex RX-3 (Velp Scientifica, Usmate, Italy) for another 2 min. The homogenate was centrifuged (Hettich, Micro 22R, Andreas Hettich GmbH & Co., Tuttlingen, Germany) at 3000 rpm for 15 min, sustaining the 40 °C extraction temperature. The supernatant was separated and kept in the dark in open trays (24 h) for acetone evaporation.

The WSE was further standardized in TPC by FC spectrophotometric assay according to a method previously described [18]. In brief, 20 µL of each sample were mixed with 100 µL of FC reagent in a 96 well plate. After 3 min, 80 µL of 7.5% sodium carbonate solution were added and the plate was incubated in the dark at room temperature for 30 min. The absorbance was measured at 760 nm against a solvent blank using a Synergy HT Multi-Detection Microplate Reader (BioTek Instruments, Inc., Winooski, VT, USA), and the TPC was expressed as mg gallic acid equivalents (GAE)/g.

### 2.3. Animals and Experimental Protocol

The experimental protocol was reviewed and approved by the Commission of Ethics of the University of Medicine and Pharmacy from Cluj-Napoca (decision no. 97/09.03.2020) and the Veterinary and Food Safety Department from Cluj-Napoca (decision no. 218/26.05.2020). The experiment was conducted in accordance with the internationally accepted principles (Directive 2010/63/EC) on the protection of laboratory animals used for scientific purposes [19]. In line with the 3Rs (Replacement, Reduction and Refinement) of humane animal research, the minimum number of animals was used for the data to attain statistical significance. As the experiment was designed to cause as little pain and suffering as possible, the laboratory animals tolerated very well the administered substances with no changes in their behavior or state of health being detected.

Healthy male Wistar rats (*n* = 24), 3 months old, were acquired from the Practical Skills and Experimental Medicine Centre of the University of Medicine and Pharmacy from Cluj-Napoca, Romania. The animals were housed in cages (Tecniplast, Italy) following a 12-h/12-h light/dark cycle, with environment temperature and relative humidity maintained at 22 ± 2 °C and 45 ± 10%, respectively, and access to standard pelleted feed (Cantacuzino Institute, Bucharest, Romania) and filtered water throughout the investigation.

#### 2.3.1. Antitussive Test

We selected 24 rats with body weight of 227.75 ± 14.60 g (mean ± SD) which were randomly divided into four groups (*n* = 6). The animals were treated for three consecutive days by gavage: group 1 (CN)—distilled water (negative control); group 2 (Cd)—codeine phosphate, 3 mg/kg b.w./day (positive control); group 3 (WSE)—10 mL WSE (containing 134 mg GAE)/kg b.w./day; group 4 (WSE 1:2)—5 mL WSE (containing 67 mg GAE)/kg b.w./day). One hour after the last oral treatment, each rat was placed in an air-tight transparent exposure chamber and exposed to citric acid aerosols (17.5%) using an ultrasonic nebulizer (Laica MD6026, Laica Spa, Italy). The antitussive effect of WSE in rats was assessed by the number and frequency of coughs caused by citric acid aerosols, cough latency after exposure being also evaluated.

The exposure period to citric acid was 4 min, followed by a further observation period of 4 min. The total number of coughs was determined over a total period of 8 min. Individual coughs were recorded with a video data acquisition platform and detected by trained staff. Each of the animals was exposed to citric acid aerosols only once.

The antitussive effect was expressed as the percentage of inhibition of number of coughs and was calculated using the equation:

% of inhibition = 100 − (Ct × 100)/C_0_

where C_0_ was the number of coughs in the negative control group and Ct was the number of coughs in the treatment group [20].

#### 2.3.2. Biological Samples

Each animal used in the antitussive test was subjected to isoflurane general anesthesia 6 h after the citric acid aerosol exposure and blood was collected from the retro-orbital sinus in the absence of anticoagulant. Afterwards, all rats were sacrificed by cervical spine dislocation and autopsied. Lungs were collected from all animals. Immediately, a lung lobe from three rats in each group was fixed in 10% neutral buffered formalin for histopathological analyses. Another lobe was frozen in liquid nitrogen and preserved at −80 °C. Lung tissue samples were weighed and homogenized with 50 mM Tris buffer 50 mM (pH 7.4) (1:5, *w*/*v*), in two stages. A crude tissue homogenate was obtained in the first step using a manual Potter-Elvehjem (Sigma-Aldrich, Schnelldorf, Germany) tissue grinder, which was then sonicated by means of an ultrasonic homogenizer (T 18; IKA Labortechnik, Staufen, Germany). The obtained tissue homogenates were used for the assay of nitric oxide (NO), reactive oxygen species (ROS), IL-6, CXC-R1, and CXC-R2. Serum was separated from blood by centrifugation after coagulation and served to determine total antioxidant capacity (TAC).

### 2.4. Determination of the Total Protein Content

The Bradford method was applied for determination of total protein content using BSA (2 mg/mL) as standard for calibration and CBB as color reagent [21]. The tissue samples were homogenized with 50 mM Tris buffer (pH 7.4) (1:10, *w*/*v*) and the absorbance was measured at 595 nm using a Jasco V-530 UV/Vis spectrophotometer (Jasco, Japan). The total protein content was used to normalize the results for oxidative stress and inflammatory biomarkers.

### 2.5. Oxidative Stress Biomarkers

#### 2.5.1. Reactive Oxygen Species

The cellular redox processes are commonly monitored using DCFH-DA. This assay was selected to measure the ability of WSE to protect against oxidative stress in the rat tissues employing a method described earlier [22]. Briefly, 10 µL of homogenate, 180 µL of PBS, and 10 µL of 1 mM DCFH-DA were mixed in a microplate well (sample processed in triplicate). The blank control was achieved by adding 190 µL PBS to 10 µL homogenate. The conversion of DCFH-DA to the fluorescent compound 2′,7′ dichlorofluorescein (DCF) was analyzed using a Synergy 2 Multi-Mode Microplate Reader at λexc = 484 nm and λem = 530 nm, and the results were conveyed as arbitrary units (AU)/mg protein.

#### 2.5.2. Nitric Oxide Level

The quantification of total NO (nitrites and nitrates) was performed using a slightly modified previously described assay [23]. The homogenates were deproteinized by adding an equal volume of acetonitrile, vortexed for 1 min and centrifuged at 5000 rpm and 4 °C for 10 min. To reduce nitrates to nitrites, a volume of 50 µL VCl_3_ (0.5% prepared in 0.5 M HCl) was added to the supernatant, followed by 50 µL Griess reagent obtained extempore from Griess I (0.33% sulphanilic acid in 15% acetic acid) and Griess II (0.066% alpha-naphthylamine in 15% acetic acid) (1:1, *v*/*v*). The absorbance of the solution was measured at 540 nm after incubation at 37 °C for 30 min. A scale of sodium nitrite standards (concentrations from 0 to 152 nmol/mL) was employed and the results were expressed as nmol/mg protein.

#### 2.5.3. Total Antioxidant Capacity by Trolox Equivalent Antioxidant Capacity (TEAC) Assay

The total antioxidant capacity (TAC) was assessed by a spectrophotometric method previously described [24]. The assay is based on the capacity of antioxidants in the solution to decolorize the blue-green ABTS radical cation according to their concentrations and antioxidant capacities. Antioxidants present in the sample accelerate the bleaching rate to a degree proportional to their concentrations. This reaction can be monitored spectrophotometrically at 660 nm and the bleaching rate is inversely related with the TAC of the sample. For the calibration curve, Trolox was used, a water-soluble analog of vitamin E, which is widely used as a traditional standard for TEAC measurement assays, and the results are expressed as mmol Trolox equivalent/L.

### 2.6. Inflammatory Biomarkers

The anti-inflammatory potential of the extract was evaluated by measuring the levels of three inflammatory markers, IL-6, CXC-R1 and CXC-R2 in lung homogenates using commercially available ELISA Kits according to the manufacturer’s instructions and further normalized to the protein content measured using the Bradford method.

### 2.7. Histopathological Analysis

The lung tissues from each group were fixed in formaldehyde for 24 h and embedded in paraffin blocks. Sections of 5 µm-thickness were obtained using a microtome (Microtec, Rotary Microtome CUT 4060, Germany), displayed on albumin treated glass slides and stained with hematoxylin-eosin (HE) for histopathological evaluation [25]. The tissue samples were processed in an autostainer (Leica TP 1020, Leica Biosystems, Wetzlar, Germany). The stained lung sections were assayed for possible toxic effects. The slides were examined using a microscope (Leica DM750, Leica Biosystems, Wetzlar, Germany) connected to a digital camera (Leica ICC50 ND, Leica Biosystems, Wetzlar, Germany), and analyzed by Leica Application Suite (LAS) V4.12 software Leica Biosystems, Wetzlar, Germany). For the objective evaluation of the histological changes, a morphometric procedure of image analysis, using the open-source platform for biological-image analysis ImageJ software (http://imagej.nih.gov/ij/), was performed on the scanned histopathological slides (Slide Converter, 3DHISTECH Ltd., Budapest, Hungary).

### 2.8. Statistical Analysis

The data are presented as mean values ± standard error mean (SEM) and, unless stated otherwise, the normally distributed result sets were evaluated using one-way analysis of variance (ANOVA). Data analyses and graphical representation were performed in SigmaPlot 11.0 computer software (Systat Software GmbH, Erkrath, Germany). Outcomes with *p* values < 0.05 were considered statistically significant.

## 3. Results

### 3.1. Antitussive Effect

**Figure 1:**
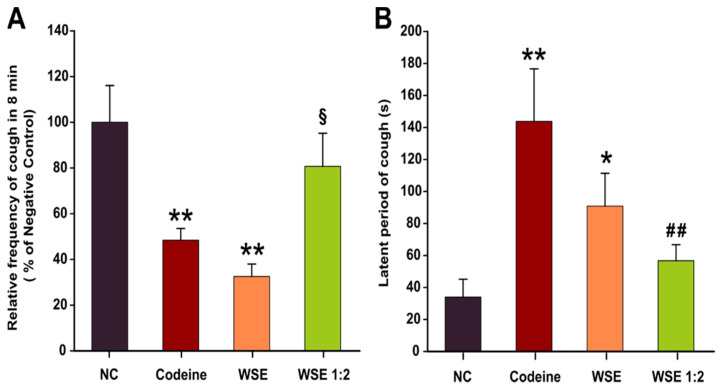
The antitussive activities of codeine, WSE, and WSE 1:2. Relative frequency of cough in 8 min (**A**) and Latent period of cough (**B**). Data are presented as the mean ± SEM (*n* = 6). * *p* < 0.05 and ** *p* < 0.01 compared to NC; ## *p* < 0.01 compared to Codeine); § *p* < 0.05 between the two concentration of WSE. Statistical significance differences in datasets were determined with one-way ANOVA with the Holm-Sidak post hoc test. NC—negative control; WSE—walnut septum extract; WSE 1:2—walnut septum extract diluted 1:2.

The citric acid aerosol-induced cough model in rats was carried out by exposing individual animals to citric acid aerosols (17.5%) for 4 min. In the control group, citric acid treatment induced a mean of 31.8 coughs in 8 min, with a latency of 34 sec (Figure 1), accompanied by nasal irritation and minor bleeding around the nostrils. In the positive control group, codeine phosphate at 3 mg/kg b.w./day significantly reduced the number of coughs to a mean of 15.4 and increased the latency to 144 sec, compared to control (*p* < 0.01). Minimal nasal irritation and bleeding was also observed in the codeine-treated group.

As shown in Figure 1 and Table 1, the tested extract demonstrated dose-dependent, potent antitussive activity, comparable with codeine. In the WSE group, for a dose of 10 mL/kg b.w./day, corresponding to 134 mg GAE/kg b.w./day, WSE significantly reduced cough frequency (*p* < 0.01) with a mean of 10.3 coughs in 8 min and prolonged the latency to 81 sec (*p* < 0.05). In the WSE 1:2 group, at 67 mg GAE/kg b.w./day, the cough frequency decreased by 20% and the latency increased to 56 sec. In addition, the animals in both groups treated with WSE showed no signs of nasal irritation and nose bleeding.

### 3.2. Oxidative Stress and Inflammatory Biomarkers

**Figure 2:**
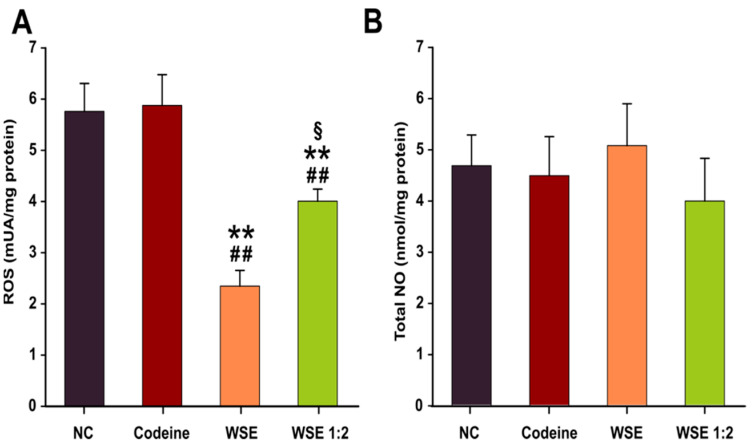
The effects of the treatments on reactive oxygen species (**A**) and total nitric oxide (**B**) in lung tissue homogenates. Values are expressed as mean ± SEM (*n* = 6). ** *p* < 0.01 compared to NC; ## *p* < 0.01 compared to positive control (Codeine); § *p* < 0.05 between the two concentration of WSE. Statistical significance differences in datasets were determined with one-way ANOVA with the Holm-Sidak post hoc test. NC—negative control; NO—nitric oxide; ROS—reactive oxygen species; WSE—walnut septum extract; WSE 1:2—walnut septum extract diluted 1:2.

**Figure 3:**
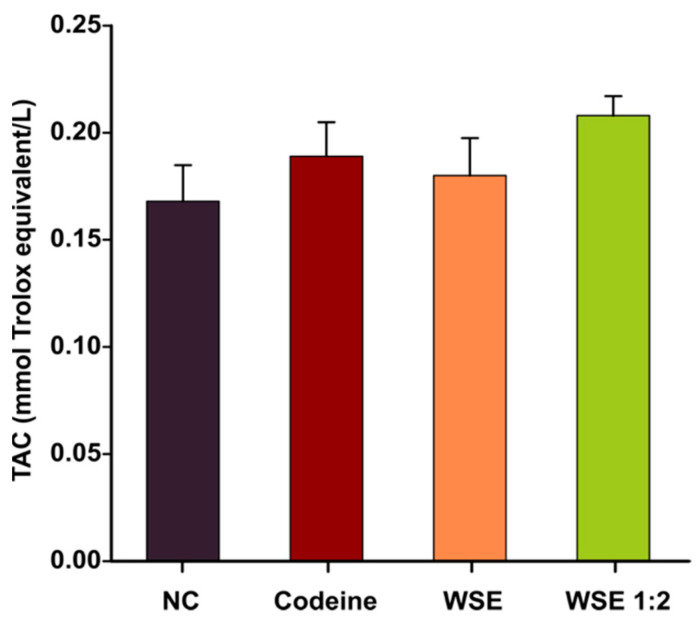
The effects of the treatments on total antioxidant capacity (TAC) in serum. Values are expressed as mean ± SEM (*n* = 6). Statistical significance differences in datasets were determined with one-way ANOVA with the Holm-Sidak post hoc test. NC—negative control; WSE—walnut septum extract; WSE 1:2—walnut septum extract diluted 1:2.

The oxidative stress and inflammatory status were assessed in lung tissue homogenates to outline a pharmacological explanation for the previously observed antitussive effects. Oxidative stress was measured using DCFH-DA, a dye that can measure ROS production in the cytoplasm as well as in intracellular organelles [26]. As shown in Figure 2B, prior treatment with WSE for 3 days, did not significantly influenced the level of total NO. However, a decrease in the quantity of ROS in lung tissue was observed using the DCFH-DA assay (Figure 2A). The decrease was dose-dependent and statistically significant in both WSE-treated groups revealing that WSE could replenish the antioxidant defense systems and reduce the risk of oxidative damage. In the case of codeine, no statistical differences were observed (Figure 2A). Regarding the TAC in serum, a statistical significance between the treatments was not observed (Figure 3). Although the latter results were not statistically significant, longer periods of supplementation might change the outcomes for this biomarker.

**Figure 4:**
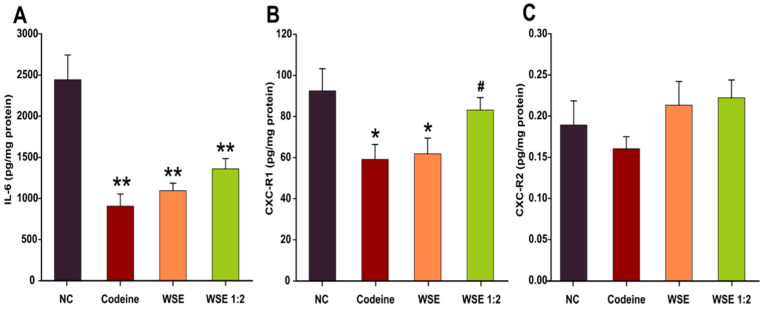
The effects of the treatments on the secretion of pro-inflammatory cytokines and receptors, IL-6 (**A**), CXC-R1 (**B**) and CXC-R2 (**C**) in lung tissue. Values are expressed as mean ± SEM (*n* = 6). * *p* < 0.05 and ** *p* < 0.01 compared to NC; # *p* < 0.05 compared to positive control (Codeine). Statistical significance differences in datasets were determined with one-way ANOVA with the Holm-Sidak post hoc test. CXC-R1—chemokine receptor type 1; CXC-R2—chemokine receptor type 2; IL-6—Interleukin 6; NC—negative control; WSE—walnut septum extract; WSE 1:2—walnut septum extract diluted 1:2.

Previous studies in humans indicated that patients with non-asthmatic chronic cough present airway inflammation with an increased sputum neutrophilia and increased levels of IL-6, IL-8, and tumor necrosis factor α (TNF-α). In this regard, the anti-inflammatory potential of the walnut septum extracts was evaluated by measuring the level of IL-6 and two receptors (CXC-R1 and CXC-R2) for IL-8 in the lung tissue homogenates. In the case of IL-6, a clear decrease in the levels of this pleiotropic pro-inflammatory cytokine was observed for codeine and WSE at both concentrations. Codeine decreased the IL-6 levels by approximately 62.5%, while WSE and WSE 1:2 decreased the levels by approximately 54% and 43%, respectively (Figure 4A). Regarding the expression of the CXC receptors, the lung tissue from the Wistar rats was more abundant in the CXC-R1 subtype (Figure 4B,C). Similar to what was observed in the case of IL-6, pre-treatment for 3 days with codeine and WSE decreased the concentration of CXC-R1 in the lung tissue homogenates (Figure 4B). The decrease was statistically significant in the case of codeine and the highest tested dose of WSE. No statically significant differences were observed in the case of CXC-R2 (Figure 4C).

### 3.3. Histopathological Analysis

**Figure 5:**
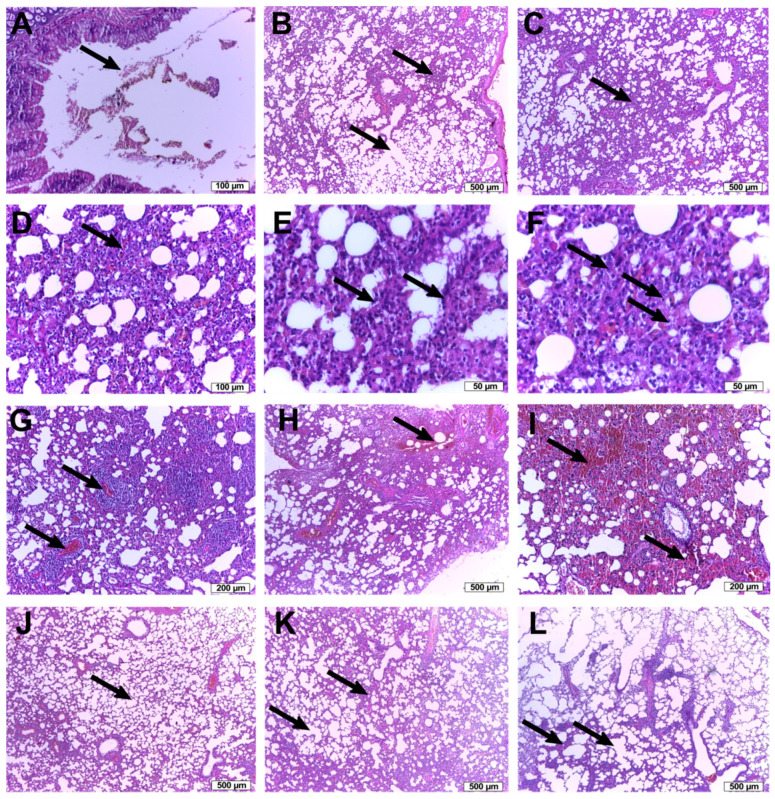
Lung tissue histopathological analysis: Negative control group (**A**–**I**): (**A**) serohemorrhagic bronchial intraluminal exudate; (**B**) collapsed alveolar septa and the emphysematous alveoli; (**C**) thickened alveolar septa; (**D**) hyperhemic alveolar capillaries with septal hypercellularity; (**E**) unflattened cubic pneumocytes; (**F**) septal macrophages and inflammatory cells; (**G**) lymphocytic infiltrate; (**H**) extensive intra-alveolar hemorrhage; (**I**) intra-alveolar red cells (hemorrhage); WSE group: (**J**) normal appearance of the respiratory parenchyma; WSE 1:2 group: (**L**) the thickened alveolar septa and emphysematous alveoli; Codeine group: (**K**) the thickened alveolar septa and emphysematous alveoli.

After the rats were sacrificed, the isolated lung tissue, carefully manipulated, without crushing, was fixed in 9% formaldehyde, subsequently included in paraffin, and stained with HE solution. Although the time between exposure to citric acid and sacrification was relatively short, histological changes in the bronchopulmonary parenchyma were observed (Figure 5), some of them severe, such as intra-alveolar hemorrhage (control group).

In the control group, the tracheobronchial tract showed focal erosions of the surface epithelium and a serohemorrhagic intraluminal exudate (Figure 5A). The lung parenchyma showed areas of normal appearance (Figure 5B), as well as areas with atelectatic alveoli along with emphysematous alveoli (Figure 5C), thus reducing the surface area available for gas exchange. Areas with thickening of the alveolar septa (Figure 5D) were also observed due to a hyperemia in the alveolar capillaries, a slight interstitial edema and a septal hypercellularity (Figure 5E). This hypercellularity can be partially explained by the lack of alveolar dilation, the alveolar spaces remaining focal small, round, therefore lined with unflattened cubic pneumocytes (Figure 5F), but also by a real increase in the number of cells in the alveolar septa (Figure 5G), with some macrophages and inflammatory cells, one case showing significant lymphocytic infiltrate (Figure 5H). Severe lesions were also observed in this group, such as extensive intra-alveolar hemorrhage (Figure 5I,J). Except the intra-alveolar hemorrhage, all the lesions previously described in the control group were also observed in the other groups exposed to citric acid, but with different intensity (Figure 5J–L).

Differences between the four groups were observed regarding the alveolar spaces, but without reaching statistical significance. In the control group, the alveolar spaces had a mean value of 11.39 ± 2.46% of the total area. The most extensive area of alveolar spaces was noticed in the group treated with WSE, with a mean value of 14.94 ± 2.64%, followed by the group treated with codeine, with a mean value of 13.17 ± 1.98% and WSE 1:2, with a mean value of 12.80 ± 3.30% of the total area.

## 4. Discussion

In folk medicine, the wooden diaphragm inside the walnut kernel has been known as an antitussive agent but, to the best of our knowledge, there is no scientific evidence to support this effect. The phytochemical analysis of walnut septum revealed a high content of bioactive compounds, especially antioxidants from the polyphenol class, such as flavonoids, ellagic acid derivatives and gallotannins, with comparable results between the studies [15,27,28].

In the current study, the antitussive, antioxidant, and anti-inflammatory potentials of an optimized WSE characterized by a high quantity of bioactive compounds were investigated in a citric acid-induced cough rodent model. The phytochemical profile of this extract was previously analyzed by liquid chromatography-mass spectrometry (LC-MS) and liquid chromatography coupled with tandem mass spectrometry (LC-MS/MS) methods (Table 2).

In our experiment, the citric acid-induced cough rodent model was obtained by exposing the animals to citric acid aerosols (17.5%) using an ultrasonic nebulizer in a closed chamber. In previous research, this model was successfully used to assess the antitussive effects of various chemical compounds or complex plant extracts from *Eriobotrya japonica* [30], *Crocus sativus* [31], and *Stemona tuberosa* [32]. Prior to exposure to the irritant, the animals were pre-treated by gavage for three consecutive days with two different doses of WSE (WSE and WSE 1:2) and a positive control (Codeine), to test a possible antitussive effect. The positive control, Codeine, significantly decreased the cough frequency by approximately 50%. Interestingly, pre-exposure to the WSE at the highest dose (134 mg GAE/kg b.w./day) resulted in a comparable effect or even superior to codeine. The antitussive effect of WSE was dose-dependent: the number of coughs decreased by approximately 68% and 20% in case of WSE and WSE 1:2, respectively, versus negative control group. In addition to lowering the cough frequency, codeine treatment prolonged the latency of coughs four-fold. Treatment with the highest tested dose of WSE significantly increased the latency of coughs, a tendency to increase this latency time being also observed for the lowest tested dose of WSE (Figure 1B). For WSE-pretreated animals, the protective effects against irritative and hemorrhagic phenomena induced by citric acid aerosols at the nasal level were obvious. This effect could be based on several mechanisms. Previous studies showed that WS extracts increase erythrocyte membrane resistance to lysis, most likely by stimulating glutathione-dependent enzyme systems [33].

Airway inflammation characterized by an increased number of immune cells in sputum and increased secretion of pro-inflammatory cytokines in airway tissues is a hallmark in patients with chronic cough. In this regard we evaluated the ability of WSE to reduce pulmonary inflammation induced by exposure to citric acid. Due to the intricate interplay between oxidative stress and inflammation, as these two processes can propagate and potentiate each other, reactive oxygen species were also measured in lung tissue homogenate. Moreover, oxidative stress was shown to contribute to airway obstruction by impairing the activation of β2-adrenoreceptors, antioxidants possessing the capacity to restore the sensitivity of these receptors to bronchodilators [34].

Pre-treatment with WSE exerted an antioxidant effect in lung tissue, decreasing ROS in a dose-dependent manner. At the highest tested dose, the ROS concentration decreased by approximately 60%, while at the lowest tested dose the decrease was approximately 30%. The ability of the WSE to mitigate oxidative stress was previously reported in an in vitro cellular model [29]. Exposure to non-cytotoxic concentrations of WSE mitigated ROS in a dose-dependent manner in different cell types (A549, T47D-Kbluc, MCF-7 and human gingival fibroblasts cells). Moreover, WSE displayed antioxidant capacity in non-stimulated and stimulated (exposure to H_2_O_2_) conditions in these in vitro models [29]. Although phytochemicals present in WSE were reported to directly neutralize ROS in in vitro studies, a short dietary supplementation may not result in a sufficient accumulation within a specific tissue to directly mitigate ROS and diminish DCFH oxidation. As in other studies [35], a more plausible explanation would be that reduced ROS concentration is related to the modulation of endogenous antioxidant defense systems. The in vivo antioxidant capacity of WSE was also reported in a D-gal-induced aging model in rats where administration of this extract for 8 weeks statistically decreased the ROS levels in brain and liver tissues [36]. The results obtained indicate that WSE possess in vitro and in vivo antioxidant potential, thus being in accordance with previously published other studies on walnut-derived extracts [37,38].

Similar to other polyphenol rich plant matrices, the observed antioxidant capacity of WSE could be explained by the content in bioactive compounds. Phytochemicals present in WSE were shown in several studies to possess antioxidant potential [39] by interacting and activating the Nrf2/ARE cellular pathway [40] that initiates the expression and synthesis of endogenous antioxidants such as glutathione [41]. Quercetin and the highest quantified quercetin glycosides in WSE, quercitrin (quercetin-3-O-rhamnoside) and isoquercitrin (quercetin 3-β-D-glucoside), were reported to possess antioxidant potential in in vitro and in vivo studies [42]. Choi et al. reported that co-exposure to quercitrin and isoquercitrin can mitigate ROS in human hepatocytes exposed to prooxidants such as 2,2′-Azobis(2-amidinopropane) dihydrochloride (AAPH), Cu^2+^ and H_2_O_2_ [43]. In addition to their direct action against ROS, quercetin and quercetin glycosides were shown to up-regulate the Nrf2/ARE pathway by repression of the Keap1 protein that inhibits the activation of this pathway [44]. A similar antioxidative potential was reported for hyperoside (quercetin 3-galactoside), exposure of human lens epithelial cells to hyperoside resulting in an increased mRNA and protein expression of heme-oxygenase-1 [45]. Other biomolecules found in WS that can be involved in the antioxidant and anti-inflammatory activities are the ellagitannins. In the gastrointestinal tract, ellagic acid, released from ellagitannins, is metabolized by the colonic microorganisms to urolithins, important modulators of oxidative stress and inflammatory action [46].

Endogenous NO plays a delicate role in homeostasis maintaining of varied cellular functions, including in the respiratory system. The local concentrations of NO are highly dynamic as the synthesis is regulated by independent enzymatic pathways. These are either constitutively expressed or induced at the gene-transcriptional level by cytokines, chemokines, and mediators. Depending on achieved local concentrations, NO can present protective respiratory effects by promoting smooth muscle relaxation and mitigating airway hyperresponsiveness to bronchoconstrictors or deleterious effects by promoting pro-inflammatory events [47]. Interestingly, NO has been shown to have a modulatory effect on inflammation, decreasing the secretion of pro-inflammatory cytokines in human alveolar macrophages challenged with bacterial lipopolysaccharides (LPS), while not altering the basal cytokine levels [48].

In the current study, pre-exposure of animals to WSE influenced NO in lung tissue homogenates of animals exposed to citric acid but not to a statistically significant extent. However, it should be noted that changes in NO were mostly reported in asthmatic patients that present eosinophilic inflammation, while no correlation between exhaled NO and other respiratory pathologies characterized by cough were demonstrated [49]. In a previous study in which we evaluated the anti-aging potential of WSE, no changes in this parameter were observed in liver and brain tissues of naturally aged animals [36]. Alternatively, these results could be due to the fact that whole lung tissues were used for the assay, and local changes of NO concentration in superficial tissue could have been masked by the bulk of the tissue. Regarding other previously reported data, a polysaccharide extract from *Opilia celtidifolia* was shown to possess bronchodilator effects in a citric acid-induced cough model, an effect partially explained by the increase in the NO production [50]. Conversely, in an ovalbumin-induced asthma model, a decrease in NO production accompanied by a down-regulation in the expression of iNOS was observed after exposure to an Orai 1 antagonist with antitussive and bronchodilator effects [51].

The anti-inflammatory potential of the WSE was further evaluated by measuring the level of IL-6 and two receptors (CXC-R1 and CXC-R2) for IL-8 in the lung tissue homogenates. Pre-treatment with WSE resulted in a decreased concentration only in IL-6 and CXC-R1. Additional studies showed that not all pro-inflammatory cytokines could be reduced by the treatment [52]. Regarding the effects observed for IL-6, we obtained similar results in an in vitro study on human gingival fibroblasts, where exposure to non-cytotoxic doses of WSE decreased the extracellular release of IL-6 in a dose-dependent manner [15]. Besides having antioxidant potential, phytochemicals present in *Juglans regia* were shown to possess an anti-inflammatory potential by inhibiting the activation of the NF-κB pathway that regulates the expression of pro-inflammatory cytokines and chemokines [53]. The complementary nature of the NF-κB and Nrf2 signaling pathways was previously reported not only for phytochemicals, but also in the case of various toxicants such as diesel exhaust particles, a concept known as the three-tiered oxidative stress paradigm [54]. The activation of the Nrf2 pathway by the polyphenols and other bioactive compounds in WSE can lead to an anti-inflammatory effect mediated by the inhibition of the NF-κB nuclear translocation [55].

In a recent study, Muzaffer et al. reported that a methanolic extract of male flowers of walnut reduced the release of pro-inflammatory cytokines IL-1 and IL-6 and TNF-α after exposure of human keratinocytes to ultraviolet B rays [38]. Moreover, different extracts from the same species, decreased the expression of pro-inflammatory adhesion molecules in isolated human endothelial cells [56]. Previous studies also demonstrated the importance of IL-8 in the development of airway diseases. Its receptors CXC-R1 and CXC-R2 are present on airway smooth muscles, being involved in the process of cell contraction with subsequent bronchial hyper-responsiveness [57]. Our data showed a decreased concentration of CXC-R1 in the lung tissue homogenates from WSE-treated animals. Thus, by reducing the risk of a chemically triggered bronchospasm, WSE could prove beneficial effects not only for cough suppression, but in larger categories of patients with airway diseases, further research being necessary to ascertain the therapeutical importance of these findings.

The anti-inflammatory effects observed in vitro are also supported by in vivo studies conducted on animals. Exposure to walnut kernel extract reduced inflammation and lung injuries in rats exposed to cigarette smoke extract [58], while exposure of mice to an extract from leaves resulted in antinociceptive and anti-inflammatory effects [59]. Moreover, Pang et al. reported that an extract isolated from the roots of *Pseudostellaria heterophylla* manifested antitussive activity via attenuation of airway inflammation by adjustment of multi-cytokine levels [52]. Similar studies on *Zataria multiflora* [60] indicated that the phytochemical compounds present in this species present a protective antitussive effect by decreasing the level of systemic cytokines such as IL-6 and IL-8 [61].

The histopathological analysis revealed small but remarkable differences between the groups. Initially, the areas represented by the thick broncho-vascular tracts were excluded, the remaining alveolar parenchyma areas being subsequently evaluated. At the level of the alveolar parenchyma, the area represented by the air spaces was quantified, as an indirect indicator of the damage/thickening of the alveolar septa or of the accumulation of fluid or blood in the alveolar spaces.

The area occupied by the alveolar spaces was the largest in the WSE group, followed by codeine and WSE 1:2 groups, while the control group presented the smallest alveolar space area relative to the total area. Therefore, even after a short treatment period, the WSE showed a positive dose-dependent effect on the alveolar space area compared to both control and codeine-treated groups. This effect could be explained by the composition of the WSE, rich in polyphenols and tocopherols. These bioactive phytochemicals revealed, as shown before, antioxidant and anti-inflammatory activities.

In our experimental model, cough was induced by an aerosolized irritative agent, citric acid, which stimulated cough-evoking chemoreceptors from the airways of exposed animals. According to previously published research, an additional inflammatory process can alter the activation profile of these receptors with a subsequent heightened sensitivity and increased transmission of stimulus to the central formations involved in cough control [62]. Thus, a reduction of inflammatory processes in the airways could represent a peripheral mechanism involved in the cough suppressant effect of WSE. However, due to the complexity of chemical composition of the extract and of physiological processes involved in cough regulation, other mechanisms could be involved in the antitussive effect of WSE.

## 5. Conclusions

This study evaluated the antitussive, antioxidant, and anti-inflammatory effects of a WSE rich in bioactive compounds, using a citric acid-induced cough model in rats. Walnut septum showed antitussive and anti-inflammatory activities and displayed potent antitussive effects.

The antitussive action of WS could be due to a cellular protective effect, by increasing the resistance of cell membranes and erythrocytes during exposure to citric acid aerosols, and an anti-inflammatory effect. These effects could be a consequence of the stimulation of the antioxidant enzyme systems and the reduction of IL-6 and CXC-R1 concentration in the lung tissue homogenates from WSE-treated animals.

Our findings provide initial scientific evidence that walnut septum could be a novel source of non-opioid antitussive drug candidates. More in-depth studies are needed in the future to clarify the molecular mechanisms responsible for the antitussive effect of walnut septum.

## Figures and Tables

**Table 1:** Effects of different treatments on the citric acid-induced cough in rats.

Group	Dose (mg/kg b.w./day)	Cough Latency ^1^ (sec)	No. of Coughs ^1^	Inhibition (%)
NC	0	34 (15–70)	31.8 (18–44)	-
Codeine	3	144 (75–200)	15.4 (11–20)	51.57
WSE	134 *	81 (60–190)	10.3 (5–15)	67.50
WSE 1:2	67 *	56 (33–100)	25.6 (11–40)	19.28

^1^ Values expressed as mean (minimum and maximum values) (*n* = 6); NC—negative control; WSE—walnut septum extract; WSE 1:2—walnut septum extract diluted 1:2. * Gallic acid equivalents.

**Table 2:** The phytochemical profile of tested WSE (determined in previously performed analytical studies on lyophilized extract).

Method (Ref.)	Chromatographic Conditions	Detection Mode	Bioactive Compound	*m/z*	mg/100 g
LC-MS [15]	Zorbax SB-C18; methanol: 0.1% acetic acid (*v*/*v*) and binary gradient at 48 °C; flow rate: 1 mL/min; injection volume: 5 µL	UV: 330 nm for 0–17 min (to detect phenolic acids); 370 nm for 18–38 min (to detect flavonoids and their aglycones) MS: ESI ^a^, negative mode, SIM ^b^ mode	Caftaric acid Gentisic acid Caffeic acid Chlorogenic acid *p*-Coumaric acid Ferulic acid Sinapic acid Hyperoside Isoquercitrin Rutoside Myricetol Fisetin Quercitrin Quercetin Patuletin Luteolin Kaempferol Apigenin	311 179 179 353 163 193 223 463 463 609 317 285 447 301 331 285 285 279	<LOD <LOQ <LOD <LOD <LOQ <LOQ <LOD 6.73 10.36 <LOD <LOD <LOD 107.31 <LOQ <LOD <LOD <LOD <LOD
LC-MS [15]	Zorbax SB-C18; methanol: 0.1% acetic acid (*v*/*v*) and binary gradient at 48 °C; flow rate: 1 mL/min; injection volume: 5 µL	MS: ESI, negative mode, SIM mode	Epicatechin Catechin Gallic acid Syringic acid Protocatechuic acid Vanillic acid	289 289 169 197 153 167	1.25 59.76 7.96 0.52 0.99 0.56
LC-MS/MS [15]	Zorbax SB-C18; methanol: acetonitrile (10:90, *v*/*v*) and isocratic elution, at 40 °C; flow rate: 1 mL/min; injection volume: 5 µL	MS: APCI ^c^, positive mode, MRM ^d^ mode	β-Sitosterol Stigmasterol Campesterol Ergosterol	397 ** 395 383 379	3101.8 <LOQ 29.21 <LOD
LC-MS/MS [29]	Zorbax SB-C18; water: methanol (7:93, *v*/*v*) and isocratic elution, at 40 °C; flow rate: 1 mL/min; injection volume: 10 µL	MS: APCI, negative mode, MRM mode	α-Tocopherol β/γ-Tocopherols δ-Tocopherol	401→386 415→400 429→163	3.35 1.73 1.47

LOD—Limit of detection (0.1 µg/mL); LOQ—Limit of quantification (0.5 µg/mL); ** [M-H_2_O+H^+^] > Main ions from spectrum (β-Sitosterol: 397 > 160.9, 174.9, 188.9, 202.9, 214.9, 243, 257, 287.1, 315.2; Stigmasterol: 395 > 255, 297, 283, 311, 241, 201; Campesterol: 383 > 147, 149, 161, 175, 189, 203, 215, 229, 243, 257; Ergosterol: 379 > 158.9, 184.9, 199, 213, 225, 239, 253, 295, 309, 323); ^a^ ESI—electrospray ionization; ^b^ SIM—selected ion monitoring; ^c^ APCI—atmospheric pressure chemical ionization; ^d^ MRM—multiple reactions monitoring.

## Abbreviations

ABTS	2,2′-azinobis-(3-ethylbenzothiazoline-6-sulfonate)
AU	arbitrary units
BSA	bovine serum albumin
b.w.	body weight
CBB	Coomassie Briliant Blue G
CXC-R1	chemokine receptor type 1
CXC-R2	chemokine receptor type 2
DCFH-DA	2′,7′-dichlorodihydrofluorescein diacetate
FC	Folin-Ciocâlteu
GAE	gallic acid equivalents
HE	hematoxylin and eosin
IL-6	interleukin 6
IL-8	interleukin 8
LC-MS	liquid chromatography-mass spectrometry
LC-MS/MS	liquid chromatography coupled with tandem mass spectrometry
LOD	Limit of detection
LOQ	Limit of quantification
NF-κB	Nuclear Factor kappa B
NO	nitric oxide
Nrf2/ARE	Nuclear factor erythroid 2-related factor 2/antioxidant response element
PBS	phosphate-buffered saline
ROS	reactive oxygen species
SD	standard deviation
SEM	standard error mean
TAC	total antioxidant capacity
TEAC	Trolox equivalent antioxidant capacity
TNF-α	tumor necrosis factor α
TPC	total phenolic content
Tris	tris(hydroxymethyl)aminomethane
Trolox	6-hydroxy-2,5,7,8-tetramethyl chroman-2-carboxylic acid
WS	walnut septum
WSE	walnut septum extract

## References

[1] Xu Y., Wang F., Guo H., Wang S., Ni S., Zhou Y., Wang Z., Bao H., Wang Y. (2019). Antitussive and Anti-inflammatory Dual-active Agents Developed from Natural Product Lead Compound 1-Methylhydantoin. Molecules.

[2] Oketch-Rabah H.A., Marles R.J., Jordan S.A., Low Dog T. (2019). United States Pharmacopeia Safety Review of Willow Bark. Planta Med..

[3] Dicpinigaitis P.V., Morice A.H., Birring S.S., McGarvey L., Smith J.A., Canning B.J., Page C.P. (2014). Antitussive Drugs—Past, Present, and Future. Pharmacol. Rev..

[4] Achan J., Talisuna A.O., Erhart A., Yeka A., Tibenderana J.K., Baliraine F.N., Rosenthal P.J., D’Alessandro U. (2011). Quinine, an old anti-malarial drug in a modern world: Role in the treatment of malaria. Malar. J..

[5] García-Estrada C., Martín J., Cueto L., Barreiro C. (2020). Omics Approaches Applied to Penicillium chrysogenum and Penicillin Production: Revealing the Secrets of Improved Productivity. Genes.

[6] Tobias J.D., Green T.P., Coté C.J. (2016). Codeine: Time to Say “No”. Pediatrics.

[7] Rusu M.E., Mocan A., Ferreira I.C.F.R., Popa D.-S. (2019). Health Benefits of Nut Consumption in Middle-Aged and Elderly Population. Antioxidants.

[8] Rusu M.E., Simedrea R., Gheldiu A.-M., Mocan A., Vlase L., Popa D.-S., Ferreira I.C.F.R. (2019). Benefits of tree nut consumption on aging and age-related diseases: Mechanisms of actions. Trends Food Sci. Technol..

[9] Alasalvar C., Bolling B. (2015). Review of nut phytochemicals, fat-soluble bioactives, antioxidant components and health effects. Br. J. Nutr..

[10] Santos A., Barros L., Calhelha R.C., Dueñas M., Carvalho A.M., Santos-Buelga C., Ferreira I.C.F.R. (2013). Leaves and decoction of Juglans regia L.: Different performances regarding bioactive compounds and in vitro antioxidant and antitumor effects. Ind. Crops Prod..

[11] Vieira V., Pereira C., Abreu R.M.V., Calhelha R.C., Alves J.M., Coutinho J.A.P., Ferreira O., Barros L., Ferreira I.C.F.R. (2020). Hydroethanolic extract of Juglans regia L. green husks: A source of bioactive phytochemicals. Food Chem. Toxicol..

[12] Dehghani F., Mashhoody T., Panjehshahin M. (2012). Effect of aqueous extract of walnut septum on blood glucose and pancreatic structure in streptozotocin-induced diabetic mouse. Iran J. Pharmacol. Ther..

[13] Ramishvili L., Gordeziani M., Tavdishvili E., Bedineishvili N., Dzidziguri D., Kotrikadze N. (2016). The effect of extract of greek walnut (*Juglans regia* L.) septa on some functional characteristics of erythrocytes. Georg. Med. News.

[14] Ravanbakhsh A., Mahdavi M., Jalilzade-Amin G., Javadi S., Maham M., Mohammadnejad D., Rashidi M.R. (2016). Acute and subchronic toxicity study of the median septum of Juglans regia in Wistar rats. Adv. Pharm. Bull..

[15] Rusu M.E., Gheldiu A.-M., Mocan A., Moldovan C., Popa D.-S., Tomuta I., Vlase L. (2018). Process Optimization for Improved Phenolic Compounds Recovery from Walnut (*Juglans regia* L.) Septum: Phytochemical Profile and Biological Activities. Molecules.

[16] Reynoso M., Brodkiewics I., Villagra J., Balderrama Coca M., Sanchez Riera A., Vera N. (2017). Evaluation of antitussive and expectorant potential of Ziziphus Mistol Fruits (Mistol). Int. J. Pharm. Sci. Res..

[17] Yang L., Jiang H., Wang S., Hou A., Man W., Zhang J., Guo X., Yang B., Kuang H., Wang Q. (2020). Discovering the Major Antitussive, Expectorant, and Anti-Inflammatory Bioactive Constituents in Tussilago Farfara L. Based on the Spectrum—Effect Relationship Combined with Chemometrics. Molecules.

[18] Rusu M.E., Fizeșan I., Pop A., Gheldiu A.-M., Mocan A., Crișan G., Vlase L., Loghin F., Popa D.-S., Tomuta I. (2019). Enhanced Recovery of Antioxidant Compounds from Hazelnut (*Corylus avellana* L.) Involucre Based on Extraction Optimization: Phytochemical Profile and Biological Activities. Antioxidants.

[19] (2010). Directive 2010/63/EU of the European Parliament and of the Council. https://eur-lex.europa.eu/legal-content/EN/TXT/PDF/?uri=CELEX:02010L0063-20190626&from=EN.

[20] Song K.J., Shin Y.J., Lee K.R., Lee E.J., Suh Y.S., Kim K.S. (2015). Expectorant and Antitussive Effect of Hedera helix and Rhizoma coptidis extracts mixture. Yonsei Med. J..

[21] Gheldiu A.-M., Popa D.-S., Loghin F., Vlase L. (2015). Oxidative Metabolism of Estrone Modified by Genistein and Bisphenol A in Rat Liver Microsomes. Biomed. Environ. Sci..

[22] Garg G., Singh S., Singh A.K., Rizvi S.I. (2017). Antiaging Effect of Metformin on Brain in Naturally Aged and Accelerated Senescence Model of Rat. Rejuvenation Res..

[23] Boşca A.B., Dinte E., Colosi H., Ilea A., Câmpian R.-S., Uifălean A., Pârvu A.E. (2015). Curcumin effect on nitro-oxidative stress in ligature-induced rat periodontitis. Rom. Biotechnol. Lett..

[24] Erel O. (2004). A novel automated direct measurement method for total antioxidant capacity using a new generation, more stable ABTS radical cation. Clin. Biochem..

[25] Grozav A., Miclaus V., Vostinaru O., Ghibu S., Berce C., Rotar I., Mogosan C., Therrien B., Loghin F., Popa D. (2016). Acute toxicity evaluation of a thiazolo arene ruthenium (II) complex in rats. Regul. Toxicol. Pharmacol..

[26] Gopalakrishnan A., Ji L.L., Cirelli C. (2004). Sleep Deprivation and Cellular Responses to Oxidative Stress. Sleep.

[27] Li L., Song L., Sun X., Yan S., Huang W., Liu P. (2019). Characterisation of phenolics in fruit septum of Juglans regia Linn. by ultra performance liquid chromatography coupled with Orbitrap mass spectrometer. Food Chem..

[28] Genovese C., Cambria M.T., D’Angeli F., Addamo A.P., Malfa G.A., Siracusa L., Pulvirenti L., Anfuso C.D., Lupo G., Salmeri M. (2020). The double effect of walnut septum extract (*Juglans regia* L.) counteracts A172 glioblastoma cell survival and bacterial growth. Int. J. Oncol..

[29] Rusu M.E., Fizesan I., Pop A., Mocan A., Gheldiu A.-M., Babota M., Vodnar D.C., Jurj A., Berindan-Neagoe I., Vlase L. (2020). Walnut (*Juglans regia* L.) Septum: Assessment of Bioactive Molecules and In Vitro Biological Effects. Molecules.

[30] Ju J., Zhou L., Lin G., Liu D., Wang L., Yang J. (2003). Studies on constituents of triterpene acids from Eriobotrya japonica and their anti-inflammatory and antitussive effects. J. Chin. Pharm. Sci..

[31] Hosseinzadeh H., Ghenaati J. (2006). Evaluation of the antitussive effect of stigma and petals of saffron (*Crocus sativus*) and its components, safranal and crocin in guinea pigs. Fitoterapia.

[32] Chung H.-S., Hon P.-M., Lin G., But P.P.-H., Dong H. (2003). Antitussive activity of Stemona alkaloids from Stemona tuberosa. Planta Med..

[33] Dzidziguri D., Rukhadze M., Modebadze I., Bakuradze E., Kurtanidze M., Giqoshvili V. (2016). The study of the immune corrective properties of greek walnut (*Juglans regia* L.) septa on the experimental model of leukopenia. Georg. Med. News.

[34] Dal Negro R.W., Wedzicha J.A., Iversen M., Fontana G., Page C., Cicero A.F., Pozzi E., Calverley P.M.A. (2017). Effect of erdosteine on the rate and duration of COPD exacerbations: The RESTORE study. Eur. Respir. J..

[35] Fu Y., Ji L. (2003). Chronic Ginseng Consumption Attenuates Age-Associated Oxidative Stress in Rats. J. Nutr..

[36] Rusu M.E., Georgiu C., Pop A., Mocan A., Kiss B., Vostinaru O., Fizesan I., Stefan M.-G., Gheldiu A.-M., Mates L. (2020). Antioxidant Effects of Walnut (*Juglans regia* L.) Kernel and Walnut Septum Extract in a D-Galactose-Induced Aging Model and in Naturally Aged Rats. Antioxidants.

[37] Muthaiyah B., Essa M.M., Chauhan V., Chauhan A. (2011). Protective effects of walnut extract against amyloid beta peptide-induced cell death and oxidative stress in PC12 cells. Neurochem. Res..

[38] Muzaffer U., Paul V., Prasad N.R., Karthikeyan R., Agilan B. (2018). Protective effect of *Juglans regia* L. against ultraviolet B radiation induced inflammatory responses in human epidermal keratinocytes. Phytomedicine.

[39] Zhang H., Tsao R. (2016). Dietary polyphenols, oxidative stress and antioxidant and anti-inflammatory effects. Curr. Opin. Food Sci..

[40] Erlank H., Elmann A., Kohen R., Kanner J. (2011). Polyphenols activate Nrf2 in astrocytes via H_2_O_2_, semiquinones, and quinones. Free Radic. Biol. Med..

[41] Zarogoulidis P., Cheva A., Zarampouka K., Huang H., Li C., Huang Y., Katsikogiannis N., Zarogoulidis K. (2013). Tocopherols and tocotrienols as anticancer treatment for lung cancer: Future nutrition. J. Thorac. Dis..

[42] Boots A.W., Haenen G.R., Bast A. (2008). Health effects of quercetin: From antioxidant to nutraceutical. Eur. J. Pharmacol..

[43] Choi S.-J., Tai B.H., Cuong N.M., Kim Y.-H., Jang H.-D. (2012). Antioxidative and anti-inflammatory effect of quercetin and its glycosides isolated from mampat (*Cratoxylum formosum*). Food Sci. Biotechnol..

[44] Tanigawa S., Fujii M., Hou D.-X. (2007). Action of Nrf2 and Keap1 in ARE-mediated NQO1 expression by quercetin. Free Radic. Biol. Med..

[45] Park J., Han X., Piao M., Oh M., Fernando P., Kang K., Ryu Y., Jung U., Kim I., Hyun J. (2016). Hyperoside Induces Endogenous Antioxidant System to Alleviate Oxidative Stress. J. Cancer Prev..

[46] Djedjibegovic J., Marjanovic A., Panieri E., Saso L. (2020). Ellagic Acid-Derived Urolithins as Modulators of Oxidative Stress. Oxid. Med. Cell. Longev..

[47] Ricciardolo F.L.M., Sterk P.J., Gaston B., Folkerts G. (2004). Nitric oxide in health and disease of the respiratory system. Physiol. Rev..

[48] Thomassen M.J., Buhrow L.T., Connors M.J., Takao Kaneko F., Erzurum S.C., Kavuru M.S. (1997). Nitric oxide inhibits inflammatory cytokine production by human alveolar macrophages. Am. J. Respir. Cell Mol. Biol..

[49] Hoyte F.C.L., Gross L.M., Katial R.K. (2018). Exhaled nitric oxide: An update. Immunol. Allergy Clin..

[50] Šutovská M., Fraňová S., Sadloňová V., Grønhaug T.E., Diallo D., Paulsen B.S., Capek P. (2010). The relationship between dose-dependent antitussive and bronchodilatory effects of Opilia celtidifolia polysaccharide and nitric oxide in guinea pigs. Int. J. Biol. Macromol..

[51] Sutovská M., Kocmálová M., Adamkov M., Výbohová D., Mikolka P., Mokrá D., Hatok J., Antošová M., Fraňová S. (2013). The long-term administration of Orai 1 antagonist possesses antitussive, bronchodilatory and anti-inflammatory effects in experimental asthma model. Gen. Physiol. Biophys..

[52] Pang W., Lin S., Dai Q., Zhang H., Hu J. (2011). Antitussive Activity of Pseudostellaria heterophylla (Miq.) Pax Extracts and Improvement in Lung Function via Adjustment of Multi-Cytokine Levels. Molecules.

[53] Wardyn J.D., Ponsford A.H., Sanderson C.M. (2015). Dissecting molecular cross-talk between Nrf2 and NF-κB response pathways. Biochem. Soc. Trans..

[54] Fizeșan I., Chary A., Cambier S., Moschini E., Serchi T., Nelissen I., Kiss B., Pop A., Loghin F., Gutleb A.C. (2018). Responsiveness assessment of a 3D tetra-culture alveolar model exposed to diesel exhaust particulate matter. Toxicol. Vitr..

[55] Ahmed S., Luo L., Namani A., Wang X., Tang X. (2017). Nrf2 signaling pathway: Pivotal roles in inflammation. Biochim. Biophys. Acta Mol. Basis Dis..

[56] Papoutsi Z., Kassi E., Chinou I., Halabalaki M., Skaltsounis L., Moutsatsou P. (2008). Walnut extract (*Juglans regia* L.) and its component ellagic acid exhibit anti-inflammatory activity in human aorta endothelial cells and osteoblastic activity in the cell line KS483. Br. J. Nutr..

[57] Govindaraju V., Michoud M.-C., Al-Chalabi M., Ferraro P., Powell W.S., Martin J.G. (2006). Interleukin-8: Novel roles in human airway smooth muscle cell contraction and migration. Am. J. Physiol. Cell Physiol..

[58] Qamar W., Sultana S. (2011). Polyphenols from *Juglans regia* L.(walnut) kernel modulate cigarette smoke extract induced acute inflammation, oxidative stress and lung injury in Wistar rats. Hum. Exp. Toxicol..

[59] Hosseinzadeh H., Zarei H., Taghiabadi E. (2011). Antinociceptive, anti-inflammatory and acute toxicity effects of *Juglans regia* L. leaves in mice. Iran. Red Crescent Med. J..

[60] Boskabady M.H., Gholami Mhtaj L. (2014). Effect of the Zataria multiflora on systemic inflammation of experimental animals model of COPD. Biomed. Res. Int..

[61] Khazdair M.R., Ghorani V., Alavinezhad A., Boskabady M.H. (2020). Effect of Zataria multiflora on serum cytokine levels and pulmonary function tests in sulfur mustard-induced lung disorders: A randomized double-blind clinical trial. J. Ethnopharmacol..

[62] Keller J.A., McGovern A.E., Mazzone S.B. (2017). Translating cough mechanisms into better cough suppressants. Chest.

